# Supplementary material for: Chromothripsis during telomere crisis is independent of NHEJ, and consistent with a replicative origin
Source: Genome Res. 2019 May;29(5):737–49. doi: 10.1101/gr.240705.118 (PMC6499312; doi:10.1101/gr.240705.118)
Supplement: Supplemental Material [file supp_gr.240705.118_Supplemental_file_1.zip › contigs/annotated_contigs/DB93/contig.2.DB93_length_268_mean_cov_4.4776119403.docx]

**DB93_length_268_mean_cov_4.4776119403**

```
GGGACTTGCCATCTTGTTGCATGTATACAGAATACATGTATACACACATGCACATATATGTATATATAAGTAGATAATTTAGCATAGGA
 >chr17:50819044-50819213 + E=1e-87
ATTGATTGGCTGAGGTGATTGTGAAAGCCAAGAGTGCCACAAATTGCCATTTGTGAGTTAGAGAACTCAGAAAAGC|CAGT|ATTTCTG
                                                                             >chr17:508
GAGAGTATTAAGTATGATAATCACTCTGCTCCTATATTCTCCTTTAATGTTTGCAATACCCAAAGGAAAATTAATGCAAGAGAAGAAAT
15715-50815818 - E=1e-44
ATA
```
